# Isolated right atriopathy and microreentry atrial tachycardia in a young male

**DOI:** 10.1002/joa3.13119

**Published:** 2024-07-28

**Authors:** Dimitrios Tsiachris, Christos‐Konstantinos Antoniou, Georgios Deligiannis, Christodoulos Stefanadis, Konstantinos Tsioufis

**Affiliations:** ^1^ First University Department of Cardiology, National and Kapodistrian University of Athens Hippokration General Hospital Athens Greece; ^2^ Pacing and Electrophysiology Laboratory Athens Heart Centre, Athens Medical Centre Marousi Greece

**Keywords:** atrial tachycardia, electroanatomical mapping, idiopathic atrial fibrosis

## Abstract

Atrial tachycardias in young patients may portend ominous prognosis. We present the case of a 17‐year‐old male with atrial tachycardia and extensive low‐voltage areas in the right atrium, its treatment, and discuss potential diagnoses.

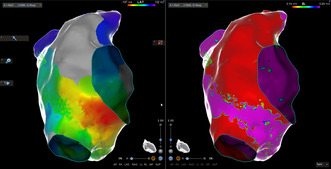

A 17‐year‐old male presented to the emergency department complaining of palpitations and tachycardia over the last few hours. An ECG revealed a fast atrial rate with 2:1 ventricular conduction and a ventricular rate of 125/1′. P wave morphology was negative in the inferior leads with a sawtooth pattern and positive in lead V1, strongly suggesting typical cavotricuspid isthmus‐dependent atrial flutter. One‐month prior, a similar episode had been successfully terminated with calcium channel blockers. There were no previous cardiac interventions, patient denied any use of illicit or recreational medications, and family history was negative for genetic heart disease or sudden death; however, there was a recent history of viral infection. Arrhythmia was now resistant to calcium channel blockers and was terminated after 36 hours of intravenous amiodarone administered only for cardioversion purposes. A cardiac magnetic resonance study was carried out the next day and revealed only increased right atrium dimensions and volumes along with the absence of findings suggesting myocarditis. It was decided to proceed with invasive three‐dimensional electroanatomical mapping (3D‐EAM) and radiofrequency ablation (RFA) of the arrhythmia.

Upon substrate mapping (Carto 3 v.7.5—PentaRay mapping catheter), and on sinus rhythm, extensive fibrosis of the anterolateral, including the appendage, and inferoposterior part of the right atrium was revealed, along with a narrow strip of normal tissue in‐between the inferior vena cava and the lateral fibrotic part, representing a potential isthmus (Figure 1). Consequently, to determine left atrial involvement, transseptal puncture and 3D‐EAM of the left atrium were performed, revealing the absence of any abnormal substrate whatsoever (Figure 2). Upon tachycardia induction with burst atrial pacing (cycle length – CL = 240 ms and same P morphology as the clinical arrhythmia), activation mapping of the left atrium revealed (Figure 2) breakthrough conduction from the right chamber at the proximal coronary sinus level and, moreover, we were unable to register the whole tachycardia CL in the chamber, leading to the diagnosis of a right atrial‐sided arrhythmia.

**FIGURE 1:**
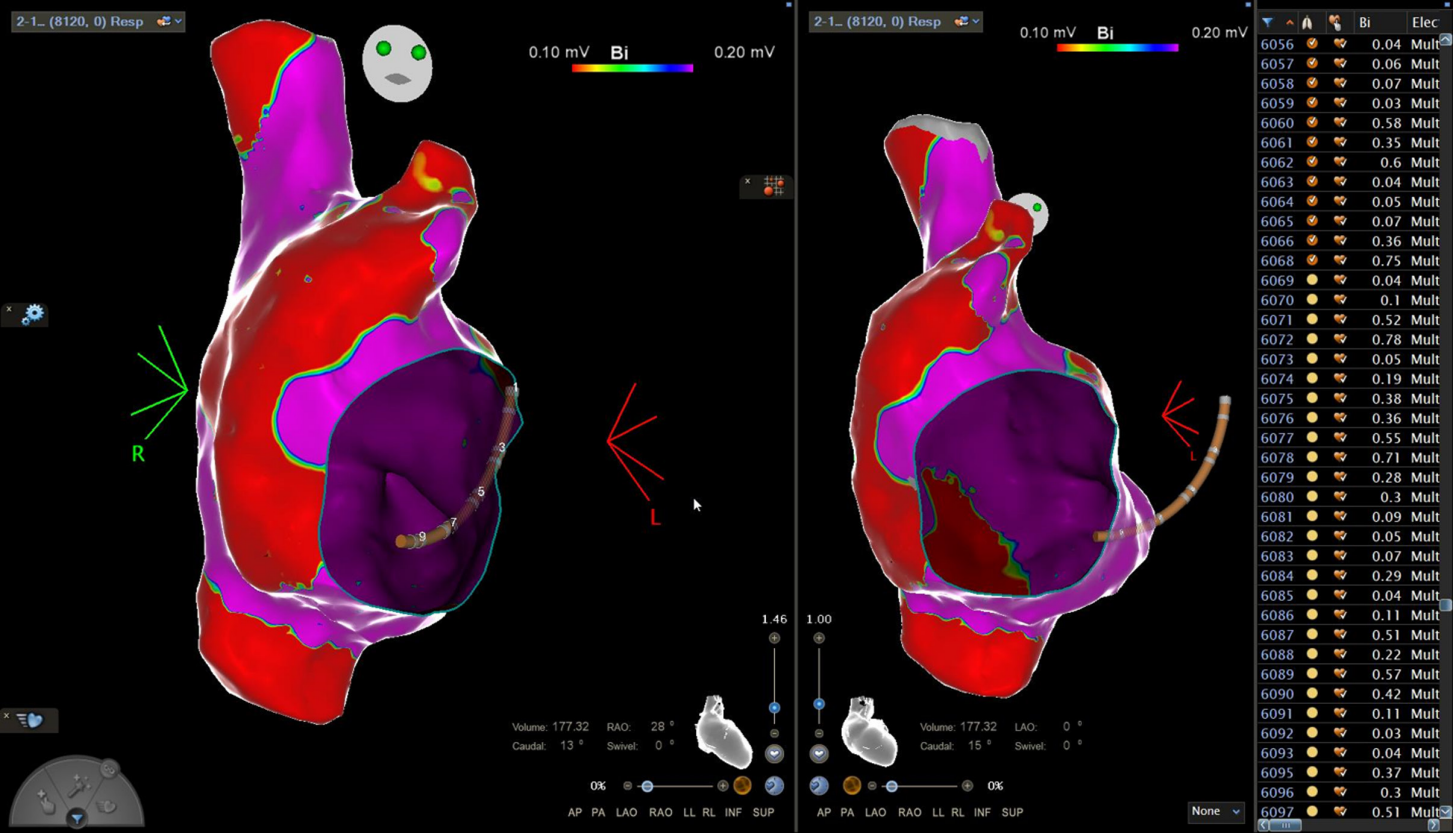
Right atrial bipolar substrate (right anterior oblique and anterior projection). A decapolar catheter in the coronary sinus and a potential isthmus are visible. Tissue proximity index was used throughout the whole mapping procedure.

**FIGURE 2:**
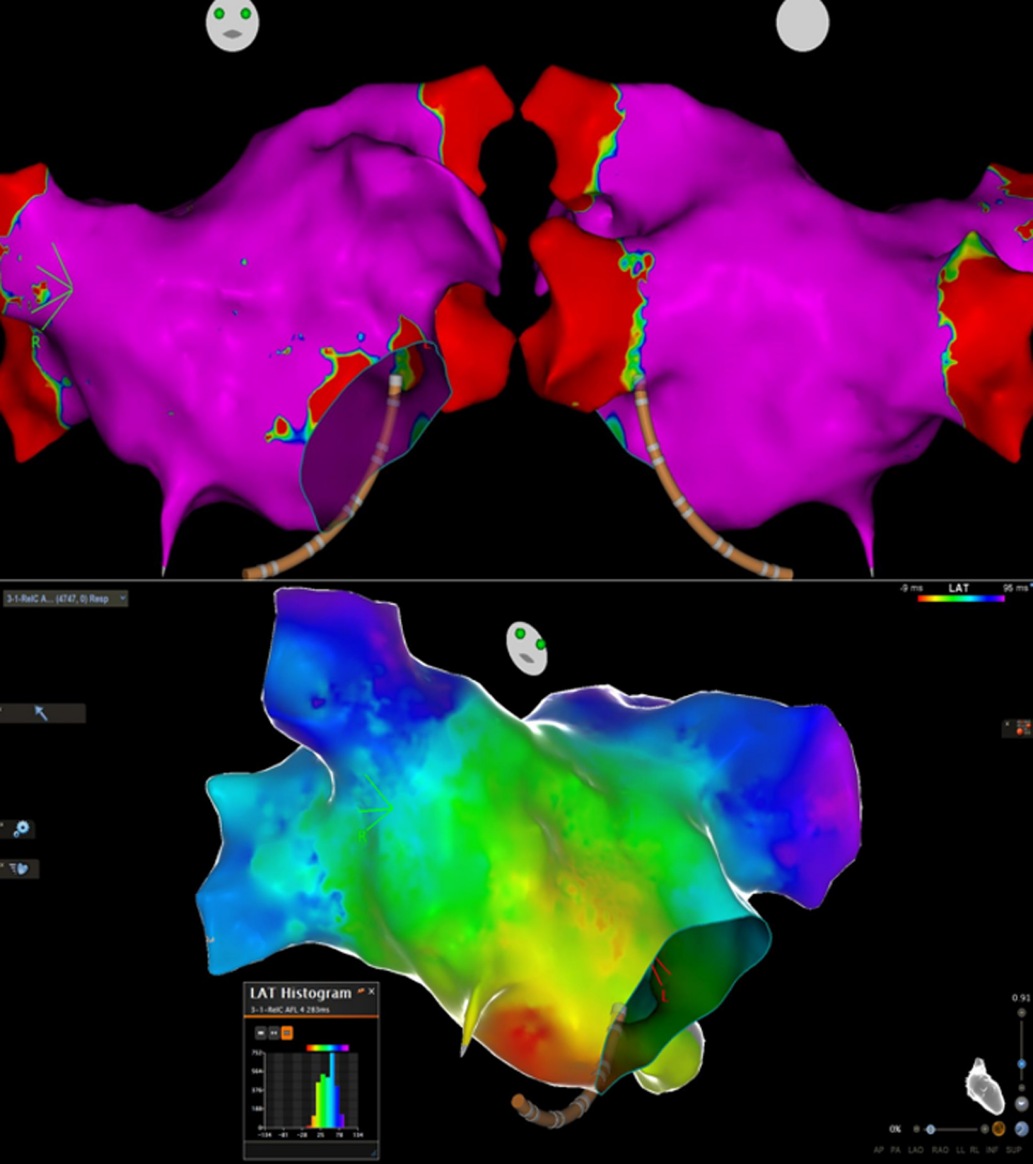
Left atrial substrate bipolar mapping – anterior and posterior view and activation mapping. Note absence of abnormal low‐voltage areas (cut‐off values for bipolar dense scar: 0.1–0.2 mV), lack of registering the whole CL even after complete mapping of the chamber and breakthrough conduction coming from the right atrium in the proximal coronary sinus area.

Activation mapping of the right atrium (Figure 3) demonstrated registration of the whole tachycardia CL, as well as a focal pattern, with centrifugal activation starting from the inferolateral tricuspid annulus and ascending towards the superior vena cava. Furthermore, a significant zone of no conduction was seen at the anterolateral wall and the appendage, coinciding with a fibrotic substrate. Lowering the dense scar threshold revealed potential channels in the earliest activation area and assumingly a potential isthmus (Figure 4). Consequent RFA lesions (Navistar Thermocool SF), during tachycardia, delivered at the zone of earliest activation successfully terminated the arrhythmia. The potential isthmus was ablated as well, to prevent future association with macro‐reentrant arrhythmogenesis (Figure 4). The patient was uneventfully discharged on the following day.

**FIGURE 3:**
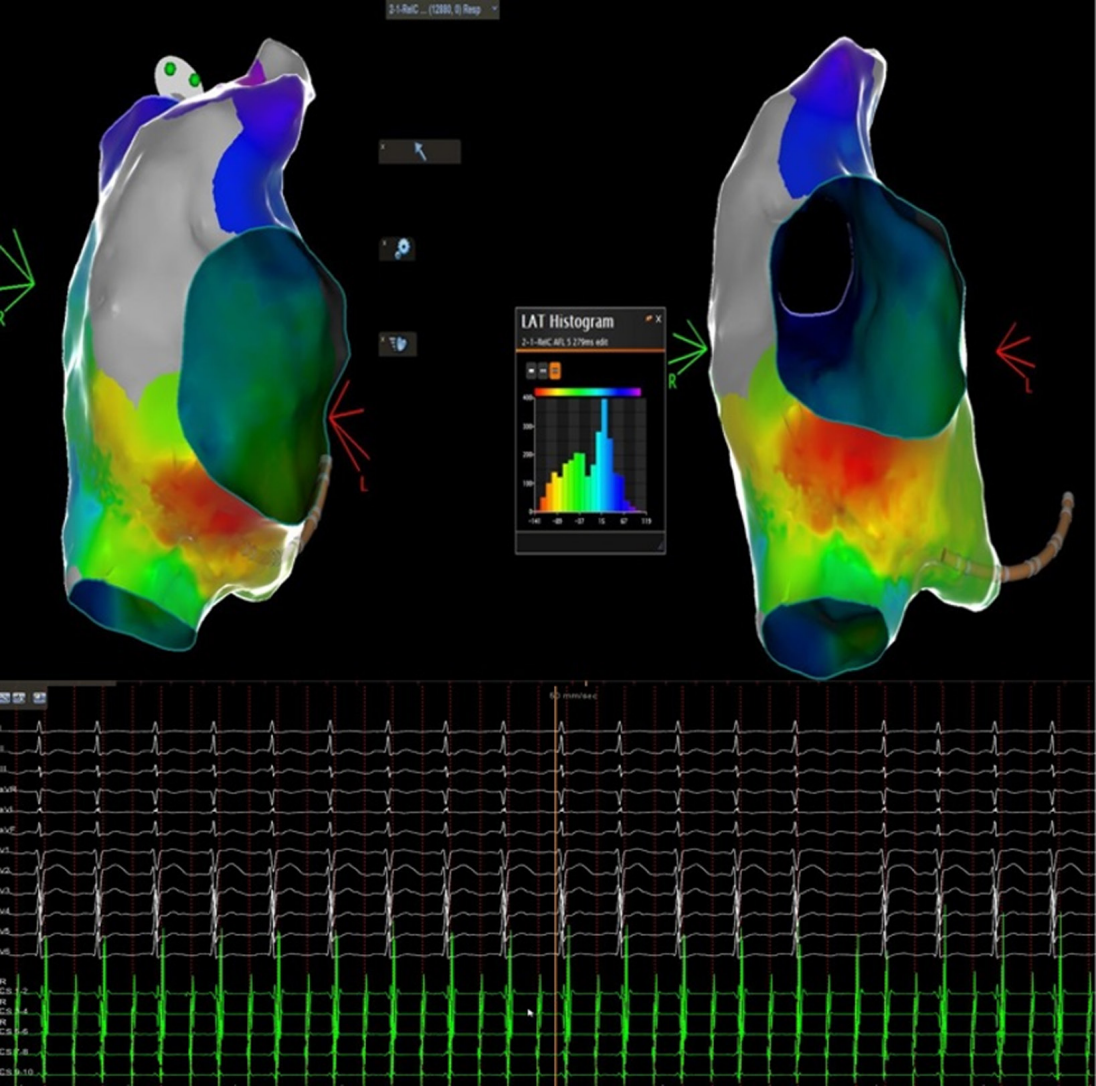
Above: Activation mapping of the right atrium. The whole CL of the tachycardia can be registered and there is a focal activation pattern at the inferolateral tricuspid annulus. Note the correspondence of the non‐conduction area with the lateral scar in the bipolar substrate map. Below: 12‐lead ECG of the tachycardia and CS decapolar catheter EGMs. Note proximal to distal activation pattern.

**FIGURE 4:**
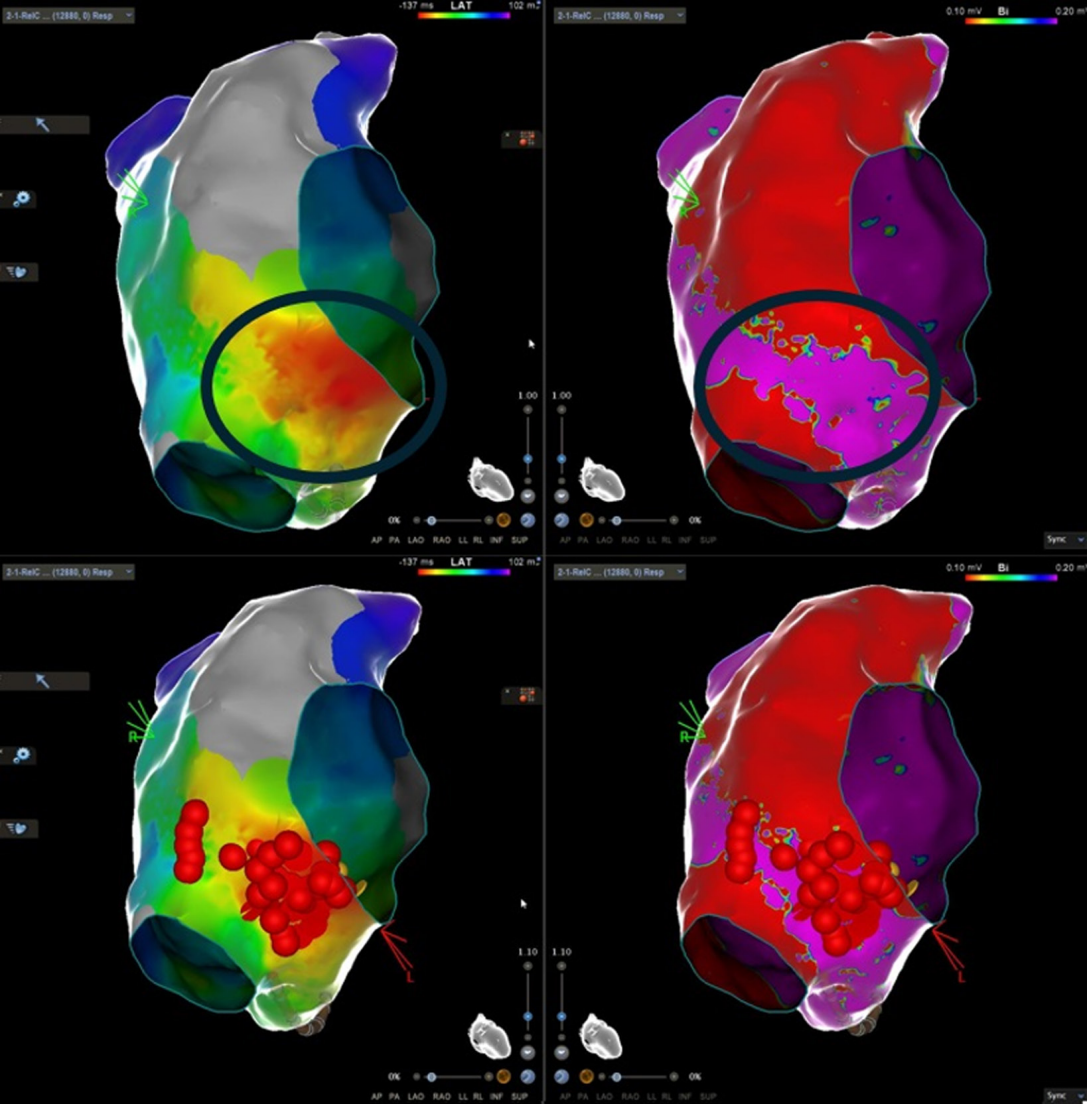
When 0.1 mV was used to demarcate dense scar (also termed area of electrical silence at this level) potential channels were revealed. Ablation lesions created in the earliest activation area terminated the arrhythmia and the potential isthmus was ablated to preclude future formation of any associated circuit.

Several conditions may be associated with the unexpected finding of isolated extensive low‐voltage substrate of the right atrium and should be included in the differential diagnosis,^1^, ^2^, ^3^, ^4^, ^5^ including atrial tachycardiomyopathy, isolated atrial myocarditis, isolated atrial amyloidosis, genetic atrial cardiomyopathies, and isolated idiopathic atrial cardiomyopathy, the latter being the most enigmatic of all the above conditions.

Atrial tachycardiomyopathy would have entailed a diffuse/patchy fibrotic pattern, not the clearly defined fibrotic areas seen in 3D‐EAM and most certainly would have involved the left atrium as well.

Acute isolated atrial myocarditis was not present in our patient, inasmuch as his inflammation markers were normal, however, we cannot indeed exclude post‐myocarditis scarring as the underlying diagnosis, even after the result of nuclear magnetic resonance imaging, given the notoriously difficult fibrosis detection in the thin atrial wall and the reported history of relatively recent viral infection.

Amyloid deposition in isolated atrial amyloidosis occurs as a consequence of increased atrial natriuretic peptide (ANP) production, which then forms the basis of the amorphous amyloid, in a clinical entity distinct from transthyretin, AA, and AL amyloidosis‐associated cardiac involvement. However, it most often involves the left atrium and is associated with atrial fibrillation in more elderly populations; our patient exhibited none of these features, thus we elected not to proceed with Tc99m DPD scintigraphy, the imaging modality of choice.

Genetic cardiomyopathies limited to atrial involvement, and leading to atrial arrhythmogenesis, are most often the result of mutations in the Natriuretic Peptide Precursor A (NPPA) protein gene. Progressive deterioration of atrial function due to advancing fibrosis corresponds with changes in associated arrhythmia, evolving from focal or macroreentrant to multifocal/microreentrant atrial tachycardias and, ultimately to atrial fibrillation. Moreover, there is biatrial involvement to the point of a complete standstill. This in turn leads to increased cardioembolic risk, irrespective of conventional risk stratification score results.

Lastly, idiopathic isolated fibrotic atrial cardiomyopathy (IIF‐ACM) is an entity that has garnered interest as the underlying condition of “lone” atrial fibrillation. Clinically, it is mostly associated with atrial fibrillation and less frequently with atrial tachycardias, often recurring post‐ablation due to substrate progression, as well as with sinus node dysfunction due to infiltration and degeneration. This entity may also lead to atrial standstill and cause thromboembolic events despite very low risk scores, due to both mechanical atrial impairment and abnormal blood circulation in the distended atria. A finding of <0.1 mV local voltage in bipolar substrate mapping constitutes electrical silence and is in fact usually located in the posterolateral region in cases of right atrial involvement, rather similar to what was observed in our case (Figure 4).

Thus, all patients with atrial tachyarrhythmias found to have significantly abnormal chamber substrate should be submitted to a rigorous workup including:

- Cardiac magnetic resonance (CMR) imaging to assess chamber dimensions, ventricular involvement and diagnose specific cardiomyopathies, including acute isolated atrial myocarditis,
- Scintigraphy to screen for atrial amyloidosis,
- Gene testing to diagnose NPPA atrial cardiomyopathy.

Patients should, in any case, be followed up to assess disease progression in terms of atrial remodeling and risk of thromboembolism, particularly if electrical silence/mechanical standstill occur. Treatment of atrial arrhythmias is rather difficult given their propensity to relapse due to the ever‐changing myocardial substrate.

## CONFLICT OF INTEREST STATEMENT

Authors declare no conflict of interests for this article.

## PATIENT CONSENT STATEMENT

Patient has consented.

## PERMISSION TO REPRODUCE MATERIAL FROM OTHER SOURCES

Granted.
